# Bar Represses dPax2 and Decapentaplegic to Regulate Cell Fate and Morphogenetic Cell Death in *Drosophila* Eye

**DOI:** 10.1371/journal.pone.0088171

**Published:** 2014-02-05

**Authors:** Jongkyun Kang, Eunbyul Yeom, Janghoo Lim, Kwang-Wook Choi

**Affiliations:** 1 Department of Biological Sciences, Korea Advanced Institute of Science & Technology, Daejeon, Korea; 2 Department of Genetics, Program in Cellular Neuroscience, Neurodegeneration and Repair, Yale University School of Medicine, New Haven, Connecticut, United States of America; 3 Graduate School of Nanoscience and Technology, Korea Advanced Institute of Science & Technology, Daejeon, Korea; Simon Fraser University, Canada

## Abstract

The coordinated regulation of cell fate and cell survival is crucial for normal pattern formation in developing organisms. In *Drosophila* compound eye development, crystalline arrays of hexagonal ommatidia are established by precise assembly of diverse cell types, including the photoreceptor cells, cone cells and interommatidial (IOM) pigment cells. The molecular basis for controlling the number of cone and IOM pigment cells during ommatidial pattern formation is not well understood. Here we present evidence that *BarH1* and *BarH2* homeobox genes are essential for eye patterning by inhibiting excess cone cell differentiation and promoting programmed death of IOM cells. Specifically, we show that loss of Bar from the undifferentiated retinal precursor cells leads to ectopic expression of Prospero and dPax2, two transcription factors essential for cone cell specification, resulting in excess cone cell differentiation. We also show that loss of *Bar* causes ectopic expression of the TGFβ homolog Decapentaplegic (Dpp) posterior to the morphogenetic furrow in the larval eye imaginal disc. The ectopic Dpp expression is not responsible for the formation of excess cone cells in *Bar* loss-of-function mutant eyes. Instead, it causes reduction in IOM cell death in the pupal stage by antagonizing the function of pro-apoptotic gene *reaper*. Taken together, this study suggests a novel regulatory mechanism in the control of developmental cell death in which the repression of Dpp by Bar in larval eye disc is essential for IOM cell death in pupal retina.

## Introduction

Cell fate specification and pattern formation are major events in development. The *Drosophila* eye consists of only a few identifiable cell types that are assembled into a highly ordered structure. The repetitive arrays of ommatidia in a compound eye provide an excellent model for studying the genetic control of cellular pattern formation. Mutations that affect the eye morphology have been extensively utilized to identify specific gene functions in different steps of eye development such as retinal determination, axial patterning, and differentiation. *Bar* is one of the first genes identified by dominant mutations that reduce the eye size [1]. Two *Bar* genes encoding similar homeodomain proteins, BarH1 and BarH2, exist in tandem repeat [2], [3]. Both genes are expressed in the similar pattern in all tissues, and they are functionally redundant [3], [4]. *Bar* gene functions during *Drosophila* eye development have been extensively studied using gain-of-function mutations, but our understanding of its loss-of-function is limited.

Retinal differentiation is initiated from the morphogenetic furrow (MF) that emerges at the posterior margin of the early third instar larval eye imaginal disc. The furrow proceeds anteriorly while columns of photoreceptor clusters are formed behind it. Retinal morphogenesis occurs in two phases. In the first phase, the R8 cells are specified as the first type of photoreceptor neurons by the proneural gene *atonal* (*ato*). Subsequently, each R8 cell recruits R2-5 cells to form a precluster. In the second phase, R1, R6, R7, and four cones cells are specified from a pool of uncommitted cells generated from the second mitotic wave, and recruited to each precluster to form a mature cluster. Bar is expressed in the nuclei of R1 and R6 photoreceptors in eye imaginal disc and in primary pigment cells during the pupal stage [3]. Consistent with this expression pattern, Bar is required for the differentiation of R1, R6, and primary pigment cells [3].

Following the formation of preclusters, cone cell fates are specified in the posterior region of eye disc. Based on the morphological defects of cone cells in the region devoid of *Bar* function [3], it has been speculated that Bar is necessary for differentiation of lens from the cone cells. Furthermore, fused and bulging ommatidia were observed in the *Bar* mutant regions [5], suggesting the presence of increased mass of non-photoreceptors in IOM space. However, since Bar is not expressed in cone cells and IOM pigment cells in the pupal retina, it is unknown how Bar functions are related to cone cell differentiation and IOM cell survival. One possibility is that Bar may be involved in differentiation of cone and IOM cells by affecting their precursor cells in earlier developmental stages. In this regard, it is important to note that in addition to R1 and R6 cells, Bar is also expressed in all undifferentiated retinal precursor cells posterior to the furrow in eye disc [6].

In third instar eye imaginal disc, the nuclei of undifferentiated precursor cells stay in the basal region while those of photoreceptors migrate apically during differentiation. For this reason, undifferentiated cells are referred here as the ‘basal cells’. Interestingly, Bar expression in these undifferentiated basal cells is essential for transcriptional repression of *ato* expression [6]. In the absence of Bar, Ato is ectopically expressed posterior to the furrow and therefore ectopic R8 cells are induced to generate a number of extra photoreceptor clusters posterior to the MF. The finding of Bar functions in the basal cells raises the possibility that Bar expression in the basal cells may have additional function in regulating the cone and pigment cell development. In the second phase of recruitment, Bar and the Runt family transcription factor Lozenge (Lz) are expressed in R1 and R6 photoreceptor cells. Prospero (Pros) is expressed in R7 and cone cells, whereas dPax2 expression is induced in the cone cells as well as primary pigment cells. It has been shown that Lz directly regulates dPax2 expression in cone cell precursors [7]. However, it is unknown whether Bar is involved in cone cell development and regulation of early cone cell marker gene expression.

In this study, we addressed the questions upon the relationships between *Bar* functions in cone cell development and IOM cell death. We show that Bar is required to repress the expression of dPax2 and Pros, thus preventing ectopic formation of excess cone cells. Interestingly, loss of Bar in the basal cells results in ectopic expression of *dpp* posterior to the MF. We show that the ectopic Dpp expression in the basal cells is not responsible for the generation of extra cone cells. Rather, its ectopic expression inhibits programmed cell death in the IOM cells. Our data suggest a novel mechanism in the control of cell death in which early repression of *dpp* expression is required to elicit developmental cell death in the subsequent developmental stage.

## Materials and Methods

### Fly stocks

The following mutant and transgenic flies were used in this study: *Df(1)B^263-20^* [3], *UAS-BarH1^M13^* [8], *dPax2-lacZ* [9] and *spa-Gal4* [9]. Other strains are described in the Flybase (www.flybase.org)

### Generation of loss-of-function (LOF) mosaic clones and misexpression studies

*Bar* LOF clones were generated using *Df(1)B^263-20^* with the FLP/FRT system [10]. First instar larvae from the cross between *yw, Df(1)B^263-20^, FRT19A/FM7* females and *w, Ubi-mRFP.nls, FRT19A, hs-FLP* males were treated for 1 hour at 37°C and incubated at room temperature until dissection. For the misexpression of Bar, progeny from the cross between *lz-Gal4* female and *UAS-BarH1^M13^* (or *UAS-BarH1-RNAi*) were cultured at 25°C.

### Histology, immunostaining and IOM cell counting

Third instar eye imaginal discs were dissected in phosphate-buffer saline (PBS) on ice, fixed in 2% paraformaldehyde-lysine-periodate fixative and stained as described [6]. Pupal retinas were dissected in PBS and processed for immunostaining as described previously [11]. The following primary antibodies were used in this study: mouse anti-Cut (1∶200; Developmental Studies Hybridoma Banks [DSHB]), mouse anti-Lz (1∶100, DSHB), mouse anti-Pros (1∶100; DSHB), rabbit anti-dPax2 (1∶200; [12]), rabbit anti-pMad (1∶2000; [13]), mouse anti-β-gal (1∶100; DSHB), mouse anti-GFP (1∶200; Sigma), mouse anti-Rough (Ro) (1∶200; DSHB), and rabbit anti-Dlg (1∶600; [14]). Rabbit anti-BarH1 antiserum (1∶500) was generated and purified as described [3].

Interommatidial cell counting was done as described previously [15]. Cell type quantification for cone and primary pigment cells was done by staining for Cut and BarH1 and scoring as described [12]. Individual cells were visualized by staining for Dlg as a membrane marker. For scanning electron microscopy, fly eyes were dehydrated in an ethanol series, critical point dried, and coated with gold-palladium.

### Quantification of the relative eye size in dorsal view

The relative eye size was analyzed from the dorsal views by using ImageJ. Since *lz>dpp* had no detectable effect on the head size, the degree of eye bulging was estimated by the horizontal length between the tip of both eyes divided by the length of dorsal head. These values were normalized to that of the *lz>GFP* control.

## Results

### Loss of *Bar* function results in excess cone and IOM cells

**Figure 1:**
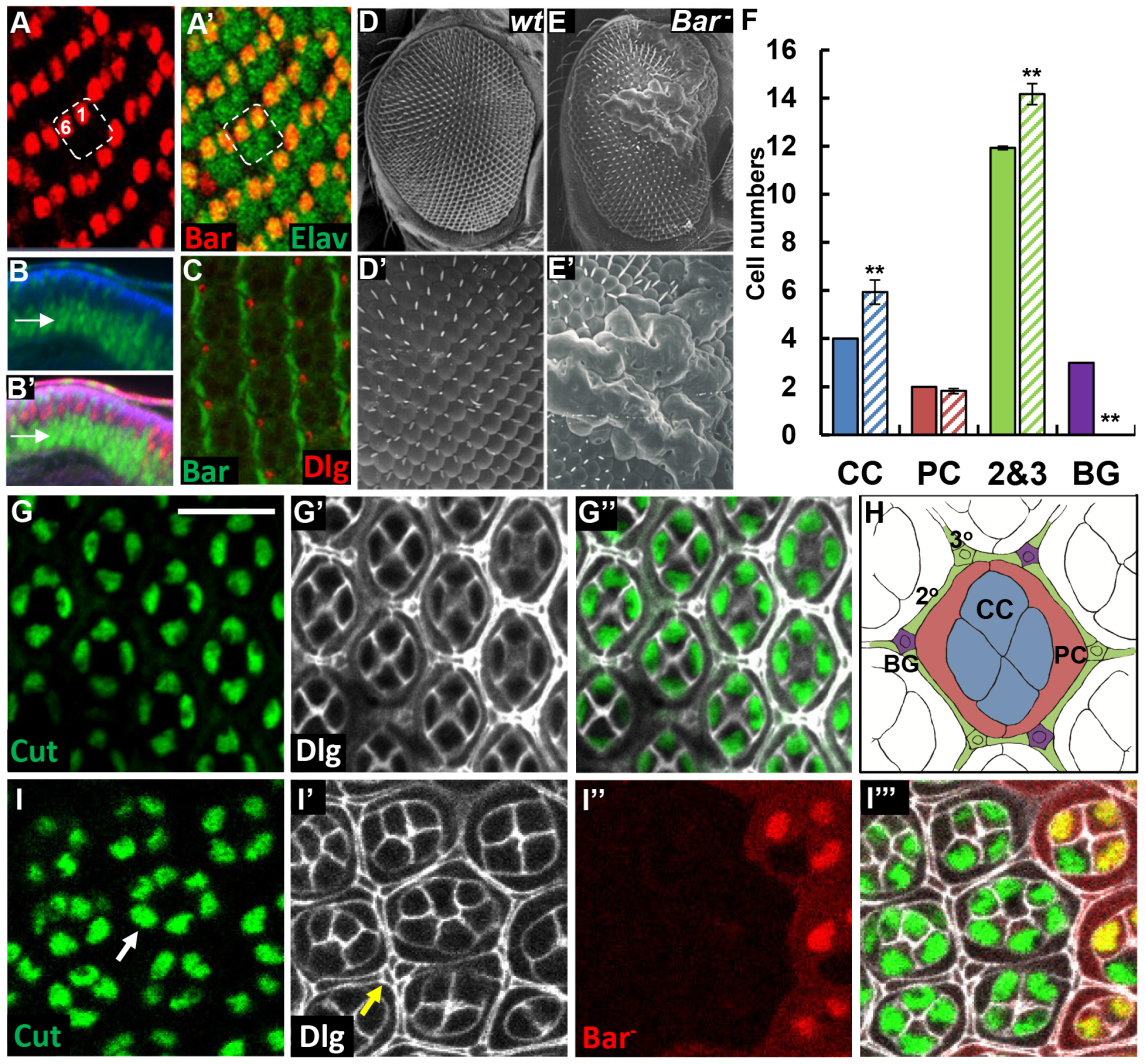
Excess cone and IOM cells in *Bar* mutant clone. (A) BarH1 (red) is specifically expressed in R1 and R6 photoreceptor cells (labeled “1” and “6” in an ommatidial cluster shown as dotted box) at 3^rd^ instar larvae stage. Photoreceptors were marked by anti-ELAV staining (green). (B) Bar is expressed in basal undifferentiated cell (arrow). Bar (green), Dlg (blue) and Ro (red). (C) Bar is expressed in primary pigment cells in pupal eye. Bar (green) and Dlg (red). (D, E) Scanning electron microscopy of adult compound eyes. (D) *w^1118^*. (E) *Bar* LOF mutant clone. D' and E' are magnified views of D and E, respectively. *Bar* LOF clones show bulged surface with fused lens. (F) Quantification of cone cells (CC), primary pigment cells (PC), secondary and tertiary pigment cells (2&3) and bristle group cells (BG) from wild-type and *Bar* LOF mutant clones in pupal eyes at 48 h APF. It shows a significant increase in the number of cone cells and IOM cells, but loss of bristle groups. (G) Wild-type pupal eye at 48 h APF stained for Cut (green; cone cell) and Dlg (grey; cell outlines). (H) Schematic presentation of different cell types in a pupal ommatidium. Different cell types are color-coded to match with the corresponding cell types shown in the panel F. (I) Pupal eye containing *Bar* LOF mutant clones at 48 h APF. *Bar* LOF clones are marked by the absence of GFP (red). Arrows in I and I' indicate extra cone cells and excess IOM cell in *Bar* mutant clone, respectively. Scale bars = 10 µm. Error bars are standard error of the mean; * P<0.05, ** P<0.01, Student's *t*-test.

*BarH1* and *BarH2* genes are functionally redundant and both genes are deleted in the deficiency *Df(1)Bar^263-20^* (Hereafter *‘Bar* mutant*’* in short). Bar is expressed in the nuclei of R1/R6 photoreceptors, undifferentiated cells posterior to the furrow in third instar larval eye disc (Fig. 1A, B) and the primary pigment cells in pupal eye (Fig. 1C). Previously, anti-proneural function of Bar has been extensively characterized using loss-of-function (LOF) *Bar* mutant clones [6], [16]. Interestingly, adult eyes containing *Bar* mutant clones show roughened external eye phenotypes. Scanning electron microscopy of such mutant clones reveals significant bulging of ommatidia and massive accumulation of fused lens materials (Fig. 1D, E). Such bulging in *Bar* LOF clones can be rescued by overexpressing wild-type *BarH1* using the *lz-Gal4,* indicating that the external eye phenotypes are due to the loss of Bar [6].

To characterize the cellular basis of the morphological defects in *Bar* mutant clones in-depth, we examined the pattern of non-neuronal accessory cells in the developing retina during pupal stages. From *Bar* LOF mutant clones generated by FLP/FRT system [10], we counted the number of cone cells, primary pigment cell, IOM cells and bristle group cells, all of which can be identified based on their shape and location in the ommatidial space. At 48 hour (h) after puparium formation (APF), each ommatidium in the wild-type eye has 4 cone cells and 2 primary pigment cells that surround the internal photoreceptor cell cluster (Fig. 1F, G & H). Individual ommatidium also contains bristles, secondary and tertiary pigment cells called IOM cells, which are shared by neighboring ommatidia. In this manner, every ommatidium has an average of 3 bristle groups at the anterior vertices, 6 secondary pigment cells at each side, and 3 tertiary pigment cells at the posterior vertices. At 48 h APF, *Bar* LOF clones showed consistently increased number of cone (5.93±0.45; about 2 extra cells/ommatidium) and IOM cells (14.17±0.44; about 2.2 extra cells) (Fig. 1F, I). The presence of extra cone cells in *Bar* LOF clones suggests that Bar is required to suppress excess cone cell formation.

During pupal eye morphogenesis, approximately 2,000 cells are eliminated by programmed cell death to establish the precise hexagonal ommatidial structure [17]. The presence of excess IOM pigment cells in *Bar* LOF clones suggests that Bar might also be required for programmed cell death in the pupal retina. In contrast to the excess number of cone and IOM pigment cells, IOM bristles are almost completely lost in *Bar* LOF clones (Fig. 1E, F & I). This indicates that Bar is required for the formation of bristle group cells. Interestingly, the number of primary pigment cells located within each ommatidium was not affected by the loss of *Bar* (Fig. 1F). Collectively, these data indicate that Bar is involved in the regulation of cell fate and cell death during differentiation of accessory cells in a cell type specific manner.

### Bar negatively regulates Pros and dPax2 expression to inhibit cone cell differentiation

**Figure 2:**
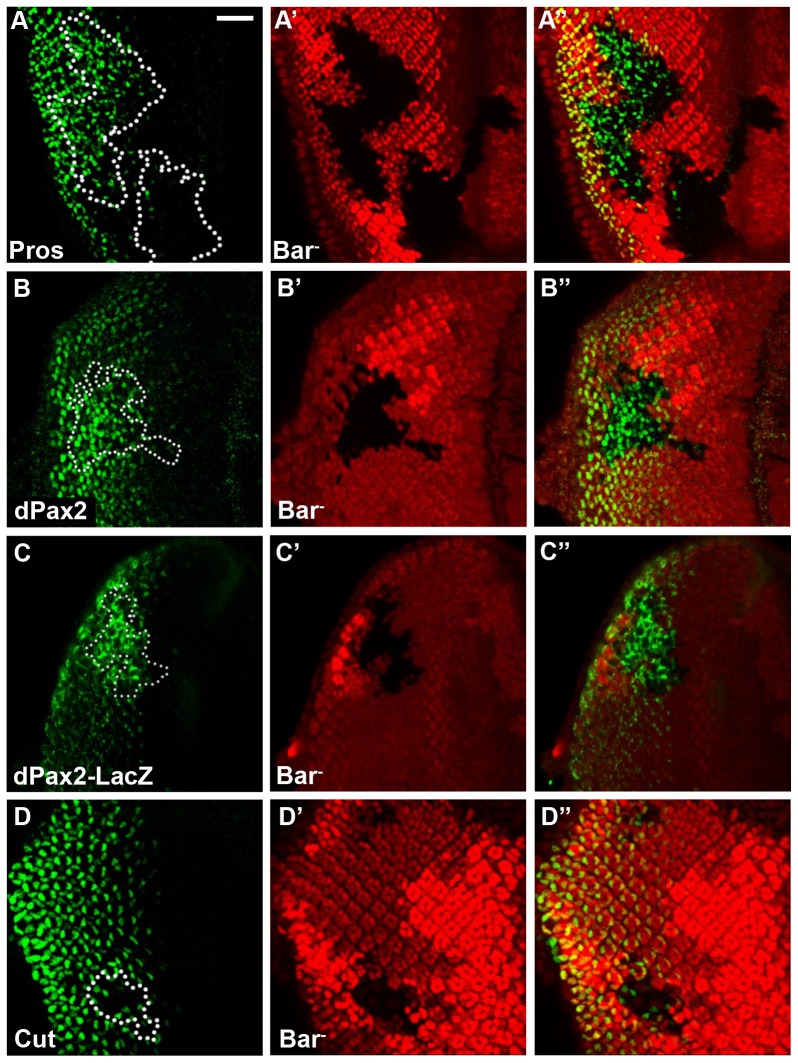
Bar regulates cone cell differentiation. Several cone cell makers were examined in *Bar* mutant clones generated from the following strain; *yw, Df(1)B^263-20^, frt19a/frt19a, ubi-mRFP, hs-flp*. The mosaic clones were marked by loss of RFP in eye discs. (A) Pros (green) was ectopically expressed in the absence of *Bar*. (B) dPax2 (green) or (C) *dPax2-lacZ* expression was ectopically induced within *Bar* LOF mutant clones. (D) Cut (green) expression was reduced in the *Bar* LOF mutant clone. Scale bar = 20 µm.

One of the most striking phenotypes of *Bar* LOF mutant clones in pupal eyes was excessive number of cone cells (Fig. 1F, I). We reasoned that this phenotype might be caused by abnormal specification of the cone cell fate in third instar larval eye disc. The cone cell fate is determined by a combinatorial activity of Pros and dPax2, and loss of either Pros or dPax2 results in a reduction of cone cells [12]. Thus, we tested the possibility of whether loss of *Bar* affects the expression of these two transcription factors. Both Pros and dPax2 were ectopically expressed in *Bar* LOF clones in the third instar eye imaginal discs (Fig. 2A, B). These results suggest that excessive cone cell formation in *Bar* LOF clones may result from ectopic expression of Pros and dPax2. We also tested whether Bar is required for the repression of dPax2 expression at the transcriptional level. *dPax2-lacZ* reporter expression was ectopically induced within *Bar* LOF clones (Fig. 2C), indicating that Bar represses transcription of *dPax2*. Interestingly, the number of cells expressing Cut, a cone cell marker [18], was reduced in *Bar* mutant clones in eye discs (Fig. 2D). Because excess Cut-positive cone cells are clearly detected in *Bar* mutant clones during pupal stages (Fig. 1F, I), the onset of Cut expression in the ectopic cone cell precursors seems to be delayed in larval eye disc. Because Bar is expressed in the basal undifferentiated cells but not in the cone cells, our data suggest that Bar is required in the basal cells, either directly or indirectly, to repress ectopic expression of dPax2 and Cut during normal eye development. To test whether Bar is sufficient to repress these genes, we overexpressed BarH1 in the developing cone cells by using *sparkling* (*spa*, synonymous with *pax2*)-*Gal4* [9]. *spa>GFP* showed normal pattern of GFP (Fig. S1A), dPax2 and Cut expression in developing cone cells (Fig. S1B). In contrast, ectopic Bar expression by *spa>BarH1* was nearly undetectable (Fig. S1D, E), suggesting that ectopic Bar expression might be downregulated or destabilized in differentiating cone cells. Despite the low level, ectopic Bar expression consistently reduced dPax2 expression (Fig. S1C). Cut expression was also slightly reduced (Fig. S1C'), as expected from the previous finding that BarH1 can reduce Cut expression [19]. Thus, Bar is necessary and sufficient for the repression of dPax2 and Cut expressions. Under the same condition, BarH1 overexpression did not show a consistent decrease in Lz expression (Fig. S1E).

### Bar is required for cell death during pupal eye development

**Figure 3:**
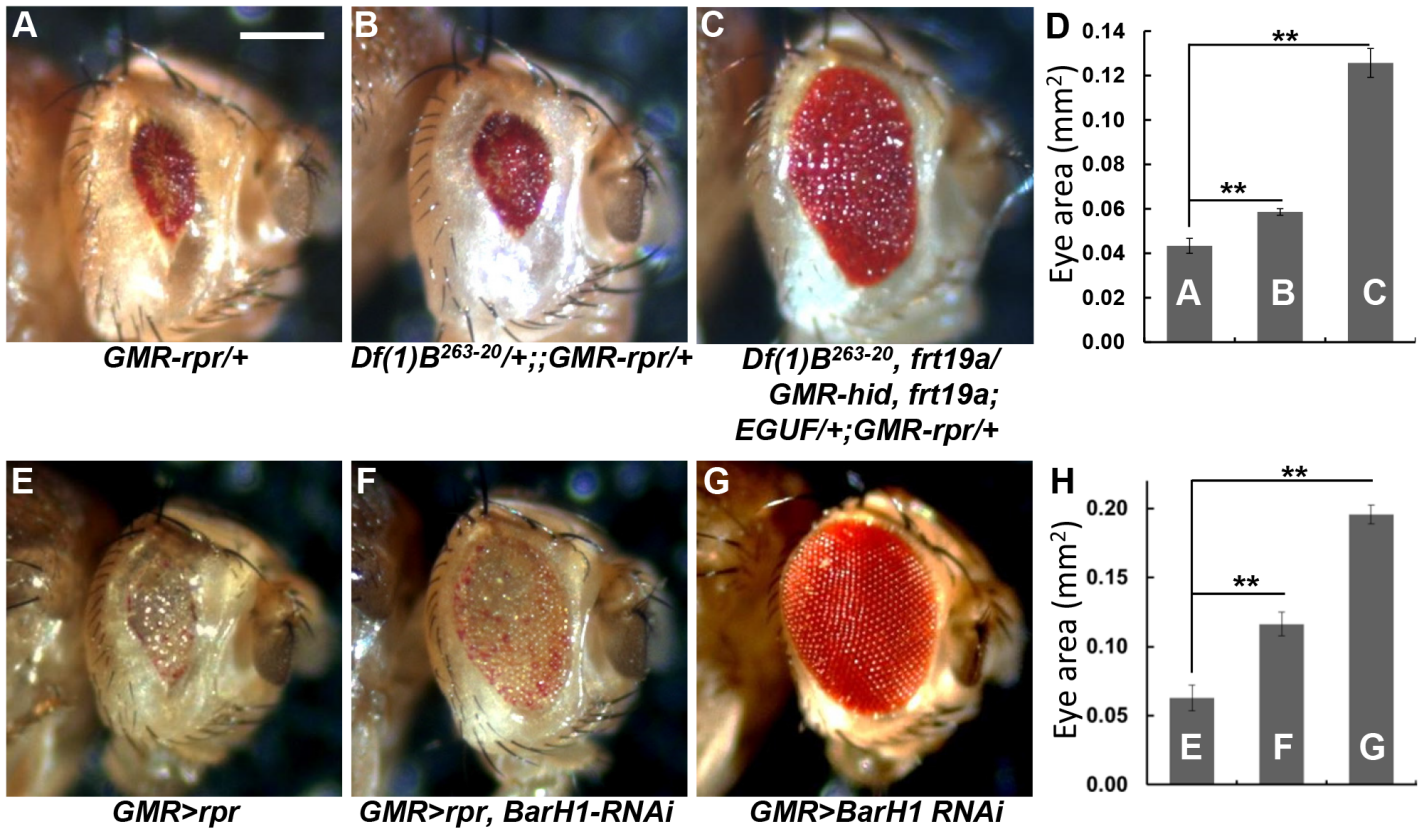
Bar suppresses programmed cell death induced by *rpr*. (A) *GMR-rpr* resulted in almost complete elimination of all retinal cells and showed small adult eye. (B) The reduction of *Bar* gene dosage (50% Bar reduction) in the *GMR-rpr* background partially restored ommatidia and eye size. (C) *Bar* LOF clones with EGUF system suppressed the effect of *GRM-rpr*. (D) Statistical analysis of eye size for (A-C). More than 15 eyes were scored for each genotype. (E) Overexpression of *rpr* gene by *GMR-Gal4* showed more severe phenotypes than *GMR-rpr*. (F) Knock-down of Bar expression by overexpressing double strand RNAi against *BarH1* (*GMR-Gal4/UAS-rpr*, *UAS-BarH1* RNAi) dramatically restored the eye size. (G) *GMR-Gal4/UAS-BarH1* RNAi showed normal eye phenotype. (H) Statistical analysis of eye size for (E-G). For G and H, error bars are standard error of the mean; ** P<0.01, Student's *t*-test. Scale Bar = 400 µm.

Extra IOM cells observed in *Bar* LOF eyes suggest that Bar may be required for IOM cell death in pupal retina. Accordingly, we examined genetic interaction between *Bar* and *reaper (rpr)* to test whether Bar is involved in the process of IOM cell death. Overexpression of the cell death gene *rpr* in the eye using the eye-specific *GMR* promoter (*GMR-rpr*) [20] eliminates most retinal cells except IOM bristles (Fig. 3A, D). Reduction of *Bar* gene dosage by half using a deletion allele *Df(1)B^263-20^* partially but consistently suppressed the small eye phenotypes of *GMR-rpr*, thus increasing the eye size by approximately 20% (Fig. 3B, D). Similar levels of suppression were found in all flies we have examined, showing 100% penetrance. To further confirm the effects of *Bar* LOF on suppressing cell death, we also used the EGUF system [21] to generate eye disc where all retinal cells except for homozygous *Bar* mutant cells were ablated using *GMR>hid* expression. Under the same condition, loss of *Bar* strongly suppressed the Rpr-dependent cell death, resulting in much larger eyes (Fig. 3C, D).

Similar to *GMR-rpr*, *UAS-rpr* expression driven by *GMR-Gal4* (*GMR>rpr*) also showed a dramatic reduction in the eye size (Fig. 3E, H). Bar RNAi knockdown at 25°C causes little effect on the external eye morphology (Fig. 3G). Under this weak RNAi condition, *Bar* RNAi strongly suppressed the cell death phenotypes caused by *GMR>rpr*, increasing the eye size by more than 2-fold (Fig. 3F, H). Since Bar RNAi itself cannot promote eye growth, the partial recovery of the eye is likely due to the suppression of the Rpr function by reduced Bar. Taken together, these results suggest that *Bar* promotes cell death by acting downstream or parallel to the *rpr* pathway.

### Bar is essential for *dpp* repression posterior to the furrow

**Figure 4:**
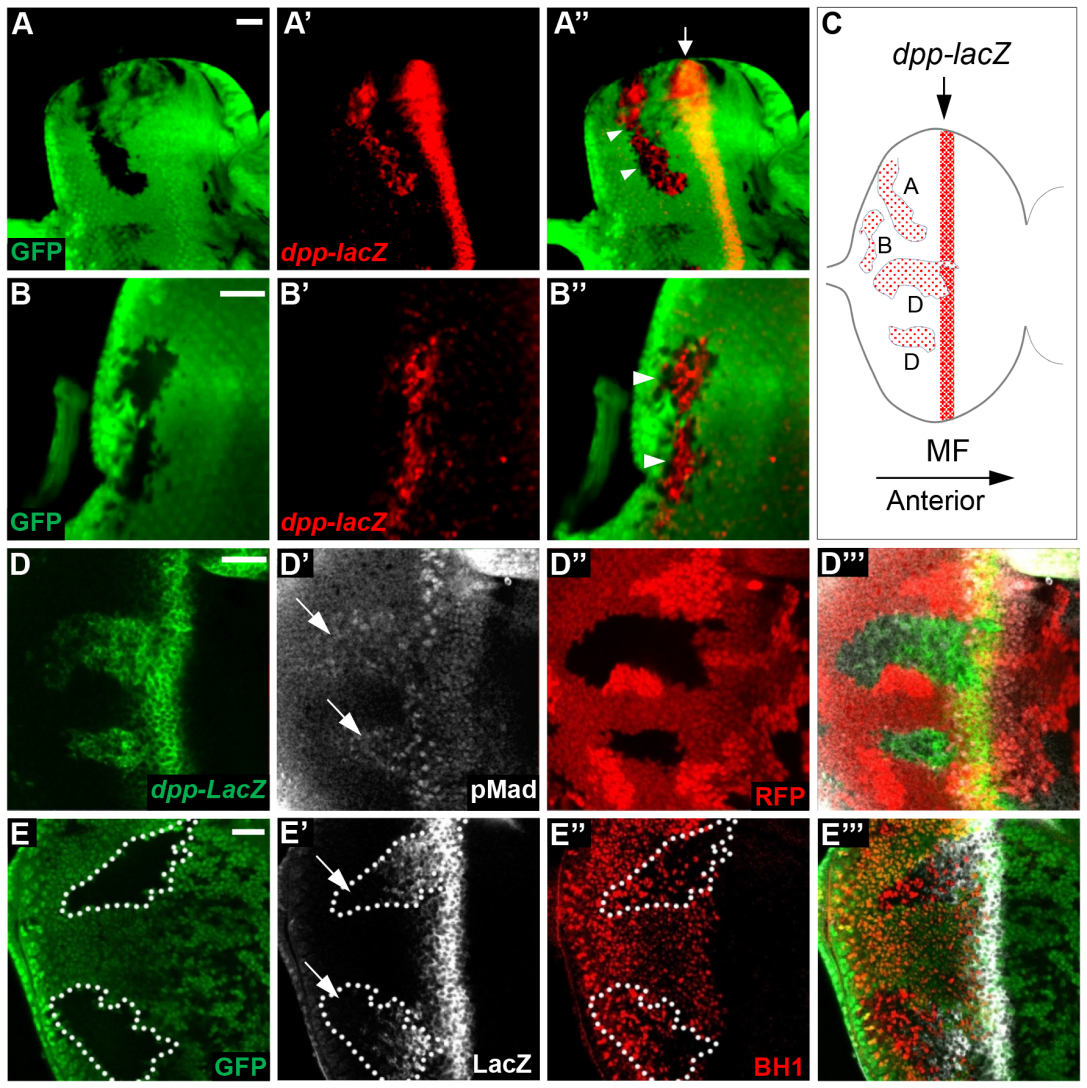
Bar is required for *dpp* repression posterior to the furrow. (A) *dpp-lacZ* (red) was ectopically induced behind the MF (arrow) within *Bar LOF* mutant clone (arrowheads) identified by the absence of GFP clone marker (green). (B) Ectopic expression of *dpp-lacZ* (red) was observed in *Bar* LOF clones near the posterior margin (arrowheads). (C) Schematic of *dpp-lacZ* expression in *Bar* LOF clones at different positions (A, B & D). (D) pMad (grey) was ectopically induced (arrows in D') behind the furrow within *Bar* LOF mutant clone identified by the absence of RFP clone marker in the larval eye discs (*Df(1)B^263-20^, frt19a/frt19a, ubi-mRFP, hs-flp; BS3.0-dpp-lacZ/+*). (E) Over-expression of wild-type *BarH1* in the basal undifferentiated cells by *lz-Gal4* strongly suppressed the ectopic expression of *dpp-lacZ*, especially in the posterior region of the *Bar* LOF clones marked by arrows, where BarH1 expression was ectopically induced. Genotype is *lz-Gal4, Df(1)B^263-20^, frt19a/frt19a, ubi-GFP, hs-flp;BS3.0-dpp-lacZ/+;UAS-dBarH1/+*. *Bar* LOF clones were marked by the loss of GFP staining. *dpp-lacZ* and BarH1 were marked by anti-β-gal (grey) and anti-BarH1 (BH1, red), respectively. Scale bars = 20 µm.

Dpp is specifically expressed in the MF in the eye disc (Fig. 4A, C, arrows). Secretion of Dpp induces the initiation and progression of the furrow, thus triggering retinal differentiation [22]. On the contrary, Bar is expressed in the undifferentiated basal cells posterior to the furrow [6]. This complementary expression pattern of Dpp and Bar raises an interesting possibility of whether these two genes regulate antagonistically to each other. It has also been shown that gain-of-function *Bar* mutations inhibit furrow progression and *dpp* expression in the furrow [23]. However, it is unknown whether Bar is required for the repression of *dpp* expression posterior to the furrow. To test whether Bar is necessary for the repression of *dpp* even at the transcriptional level, we examined the expression of an eye-specific *dpp* reporter *BS3.0 dpp-lacZ* [24] in *Bar* LOF mutant clones at different locations. Loss of *Bar* in all mutant clones (37 clones observed in 13 different eye discs) resulted in ectopic induction of *dpp-lacZ* expression behind the furrow (Fig. 4A-C). Similar ectopic expression of *dpp-lacZ* was also detected in the clones generated near the posterior margin or the equator of eye imaginal discs (Fig. 4B), as schematically shown in Fig. 4C. This suggests that Bar is required for *dpp* repression in the entire region posterior to the furrow. Next, we tested whether ectopic *dpp* expression in *Bar* LOF clones are functional, using phosphorylated Mad as a marker for active Dpp signaling [13]. We found that pMad expression level was significantly enhanced in *Bar* LOF clones (Fig. 4D', arrows). Finally, ectopic expression of wild-type *BarH1* in the basal undifferentiated cells by *lz-Gal4* strongly re-suppressed the enhanced *dpp-lacZ* expression within the *Bar* LOF clone (Fig. 4E', arrows). Taken together, Bar expression in the basal undifferentiated cells is necessary and sufficient to repress *dpp* expression posterior to the furrow.

### Ectopic Dpp expression affects IOM cell death but not cone cell fate

**Figure 5:**
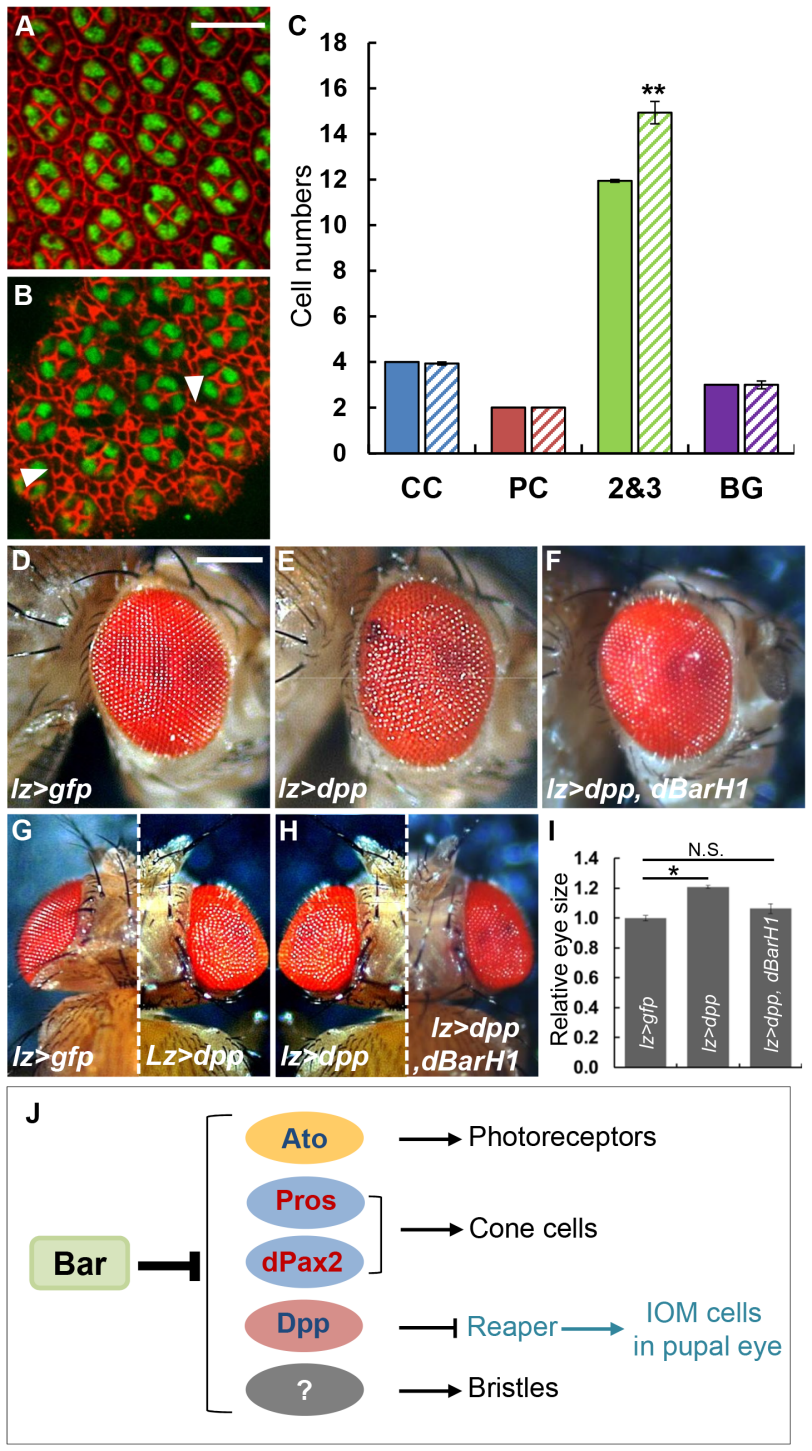
*dpp* overexpression results in excess IOM cells. (A-B) Pupal retinas at 24 h APF stained with anti-Dlg (red; cell boundary marker) and anti-Cut (green; cone cell marker). (A) Pupal retina from *w^1118^* shows a normal ommatidium structure. Normal eye with GFP expression by *lz-Gal4* was shown. Scale bar = 10 µm. (B) *lz>dpp* (*lz-Gal4/+;UAS-dpp/+*) eye showed an increased number of IOM cells (arrowheads). Note that the number of cone cells was not changed. (C) Statistical analysis of total number of IOM cells. Error bars are standard error of the mean; **P<0.01, *t-test*. (D) Normal eye phenotype of *lz>gfp* (*lz-Gal4/+; UAS-gfp/+*). (E) Dpp overexpression by *lz-Gal4* caused roughened and bulged eye. Scale bar = 200 µm. (F) Co-overexpression of wild-type *BarH1* suppressed bulged eye phenotypes of the *lz>dpp*. (G) Comparison of dorsal eye views of (D) and (E). (H) Comparison of dorsal views of (E) and (F). (I) Relative eye size measured from the dorsal view (see Materials and Methods). The bulged eye phenotype of *lz>dpp* was rescued by co-overexpressing *BarH1*. Error bars are standard error of the mean; *P<0.05, *t-test*. N.S. (Not Significant). (J) Proposed model for the role of Bar in the regulation of cell fate and morphogenetic cell death (see Discussion). Note that Bar is required for the repression of the indicated genes in the undifferentiated basal cells, and it is unknown whether the repression is direct.

Since *Bar* LOF clones induce extra cone cells as well as ectopic Dpp expression, we asked whether the formation of additional cone cells might be a consequence of ectopic Dpp expression. To address this question, we overexpressed Dpp in the basal undifferentiated cells by using *lz-Gal4*. This Dpp overexpression (*lz>dpp*) did not alter the pattern of phospho-Histone H3 staining and the arrays of photoreceptor clusters in eye disc (data not shown), indicating that it does not cause excess cell proliferation or significant defects in retinal differentiation in larval eye disc. We then examined whether it could induce extra cone cell differentiation. In wild-type pupal eye at 24 h APF, ommatidial cells are precisely organized into hexagonal arrays in which accessory cells including 4 cone cells can be recognized (Fig. 5A). In contrast, eyes with Dpp overexpression showed irregular ommatidial arrays (Fig. 5B). However, the majority of ommatidia contained 4 Cut-positive cone cells. No ommatidia showed any excess number of cone cells (Fig. 5C). Instead, some ommatidia (6.3±1.1%) showed even less than 4 cone cells. These results suggest that ectopic Dpp expression is not responsible for the formation of excess cone cells seen in *Bar* LOF clones, although it causes irregular ommatidial arrays (Fig. 5B, C).

Next, we asked whether the presence of extra IOM cells is related to the ectopic expression of Dpp in *Bar* LOF eye disc. When Dpp was overexpressed with *lz-Gal4* in the basal cells of eye disc and primary pigment cells of pupal eye, it resulted in bulging and roughening in adult eyes, consistent with the presence of excess cells (Fig. 5B, E, H & I). To find the relationship between this bulged eye phenotype and reduced cell death in IOM cells, we examined pupal eyes of *lz>dpp*. In the *lz>dpp* pupal eyes, there was an increase in IOM cells at 24 h APF (Fig. 5B, C). Furthermore, the bulged eye phenotype of *lz>dpp* was suppressed by co-overexpressing *BarH1* (Fig. 5F, H & I). Taken together, these results suggest that Bar is required for transcriptional repression of *dpp*, and the ectopic Dpp expression promotes the survival of IOM cells during pupal eye development.

## Discussion

This study supports that Bar acts as a negative regulator of cone cell formation by antagonizing the expression of dPax2 and Pros. Further, Bar is required for achieving the proper level of IOM cell death in the pupal eye, providing a permissive condition for IOM cell death in pupal eye by repressing Dpp expression in larval eye disc. In addition to the negative regulation of cone cell differentiation and *dpp* expression, Bar is essential for the formation of bristle groups. Thus, together with its anti-proneural function [6], we propose that Bar expression in the basal undifferentiated cells plays multiple regulatory roles for differentiation or patterning of most cell types posterior to the MF (Fig. 5J). It has been suggested that Lz regulates Bar expression [25]. Interestingly, although both Lz and Bar are expressed in the same basal cells, Bar expression in these basal cells appears to be independent of Lz [16]. While Lz promotes cone cell differentiation, Bar seems to be required to maintain the undifferentiated state of the basal cells. Because Bar is expressed in the basal cells but not in cone cells, the normal function of Bar is to repress ectopic dPax2 expression in undifferentiated cells. In addition, ectopic overexpression of Bar in developing cone cells can partially repress dPax2 and Cut. Our finding of ectopic *dpp* induction in *Bar* LOF clones raises the possibility that the formation of extra cone cells might be related to the ectopic *dpp* expression. However, misexpression of *dpp* in the *lz*-expressing cells did not increase cone cell number in the presence of Bar expression, suggesting that the ectopic cone cell formation in *Bar* LOF clones may be due to the derepression of dPax2, but not by Dpp. However, we cannot completely rule out the possibility of ectopic Dpp contribution to extra cone cell formation in the absence of Bar.

The excess IOM cells present in *Bar* LOF mutant clones is an indicative of reduced developmental IOM cell death. We show that reduction or loss of Bar suppresses cell death induced by Rpr (Fig. 3). This genetic interaction suggests that Bar acts downstream to Rpr, possibly involved in mediating the apoptotic function of Rpr. Our data indicate that Bar is required for transcriptional repression of *dpp* posterior to the furrow (Fig. 4A-D). Further, ectopic expression of *dpp* in the basal cells results in bulging of the eye tissues, and such bulging can be suppressed by overexpression of *Bar* in the basal cells (Fig. 5G-I). These results raise the possibility that the repression of *dpp* by Bar in the basal cells may provide a necessary condition for the apoptotic elimination of excess IOM cells that are not recruited to the ommatidia.

Death of IOM cells is initiated in an early pupal stage and peaks at a mid-pupal stage. However, Dpp expression turns off immediately posterior to the furrow in the larval eye disc. These led us to propose that immediate repression of *dpp* in the larval eye disc is important for subsequent IOM cell death during early pupal stage. Our data suggest that ectopic Dpp expression posterior to the furrow antagonizes the Rpr-dependent cell death pathway. It is worth noting that the cell death inhibitor DIAP1 directly binds the ring domain of Thickvein (Tkv), the type I receptor for Dpp [26], although its function *in vivo* has not been tested. Since Dpp can function as a cue for cell survival in wing development [27], ectopically expressed Dpp in *Bar* LOF cells may activate its cell survival signaling by binding to Tkv, which may in turn inhibit cell death by activating DIAP1 and/or DIAP2. Interestingly, it has been shown that Dpp is expressed later in primary pigment cells in the pupal retina. This Dpp expression in the primary pigment cells is required for cell shape changes during IOM cell morphogenesis [28], rather than cell survival. Thus, we propose that Dpp must be repressed posterior to the MF in larval eye disc to inhibit unnecessary cell survival signaling in the IOM cells, thus allowing cell death to occur in pupal stage. It would be interesting to see whether such temporally coordinated cell survival/death signaling is widely used for developmental cell death programs in other organisms.

## Supporting Information

Figure S1 **Effects of BarH1 overexpression on dPax2, Cut and Lz.** (A-A’’) GFP is expressed in cone cells by *spa-Gal4*. (B-B’’) As a control, GFP is overexpressed using *spa-Gal4,* and it shows normal pattern of dPax2 and Cut expression in developing cone cells. (C-C’’) In the developing cone cells, BarH1 is overexpressed by *spa-Gal4.* The level of dPax2 is significantly reduced (C). Cut staining is also weakened (C’). (D-D’’) *spa>GFP* shows normal pattern of Bar and Lz expression. (E-E’’) BarH1 overexpression does not show significant reduction of Lz expression. Scale bar = 20 µm.
(TIF)
