# Supplementary figures and images for: Bar Represses dPax2 and Decapentaplegic to Regulate Cell Fate and Morphogenetic Cell Death in Drosophila Eye
Source: PLoS One. 2014 Feb 5;9(2):e88171. doi: 10.1371/journal.pone.0088171 (PMC3914906; doi:10.1371/journal.pone.0088171)

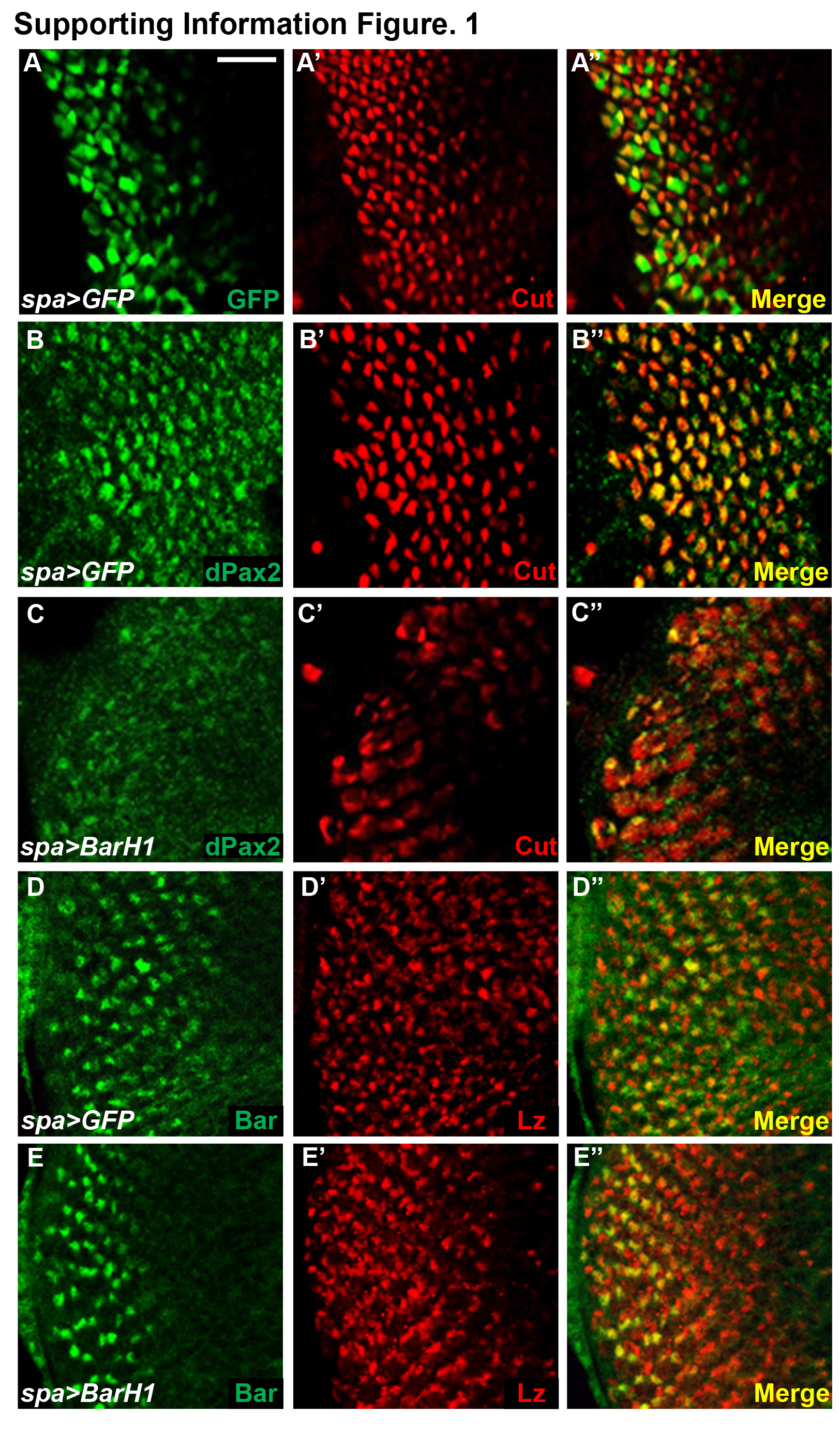

Supplement: Figure S1 — Effects of BarH1 overexpression on dPax2, Cut and Lz. (A-A’’) GFP is expressed in cone cells by spa-Gal4. (B-B’’) As a control, GFP is overexpressed using spa-Gal4, and it shows normal pattern of dPax2 and Cut expression in developing cone cells. (C-C’’) In the developing cone cells, BarH1 is overexpressed by spa-Gal4. The level of dPax2 is significantly reduced (C). Cut staining is also weakened (C’). (D-D’’) spa>GFP shows normal pattern of Bar and Lz expression. (E-E’’) BarH1 overexpression does not show significant reduction of Lz expression. Scale bar = 20 µm. (TIF) [file pone.0088171.s001.tif]
